# The impact of charge transfer and structural disorder on the thermoelectric properties of cobalt intercalated TiS_2_ †
†Electronic supplementary information (ESI) available: Powder X-ray diffraction data, thermal analysis data and additional magnetic and transport property data. See DOI: 10.1039/c5tc04217h



**DOI:** 10.1039/c5tc04217h

**Published:** 2016-02-02

**Authors:** Gabin Guélou, Paz Vaqueiro, Jesús Prado-Gonjal, Tristan Barbier, Sylvie Hébert, Emmanuel Guilmeau, Winfried Kockelmann, Anthony V. Powell

**Affiliations:** a Department of Chemistry , University of Reading , Whiteknights , Reading RG6 6AD , UK . Email: a.v.powell@reading.ac.uk; b Laboratoire CRISMAT , UMR6508 CNRS ENSICAEN , 6 bd Marechal Juin , 14050 Caen Cedex 4 , France; c STFC , Rutherford Appleton Laboratory , ISIS Facility , Didcot OX11 0QX , UK

## Abstract

A family of phases, Co_*x*_TiS_2_ (0 ≤ *x* ≤ 0.75) has been prepared and characterised by powder X-ray and neutron diffraction, electrical and thermal transport property measurements, thermal analysis and SQUID magnetometry. With increasing cobalt content, the structure evolves from a disordered arrangement of cobalt ions in octahedral sites located in the van der Waals' gap (*x* ≤ 0.2), through three different ordered vacancy phases, to a second disordered phase at *x* ≥ 0.67. Powder neutron diffraction reveals that both octahedral and tetrahedral inter-layer sites are occupied in Co_0.67_TiS_2_. Charge transfer from the cobalt guest to the TiS_2_ host affords a systematic tuning of the electrical and thermal transport properties. At low levels of cobalt intercalation (*x* < 0.1), the charge transfer increases the electrical conductivity sufficiently to offset the concomitant reduction in |*S*|. This, together with a reduction in the overall thermal conductivity leads to thermoelectric figures of merit that are 25% higher than that of TiS_2_, *ZT* reaching 0.30 at 573 K for Co_*x*_TiS_2_ with 0.04 ≤ *x* ≤ 0.08. Whilst the electrical conductivity is further increased at higher cobalt contents, the reduction in |*S*| is more marked due to the higher charge carrier concentration. Furthermore both the charge carrier and lattice contributions to the thermal conductivity are increased in the electrically conductive ordered-vacancy phases, with the result that the thermoelectric performance is significantly degraded. These results illustrate the competition between the effects of charge transfer from guest to host and the disorder generated when cobalt cations are incorporated in the inter-layer space.

## Abstract 2

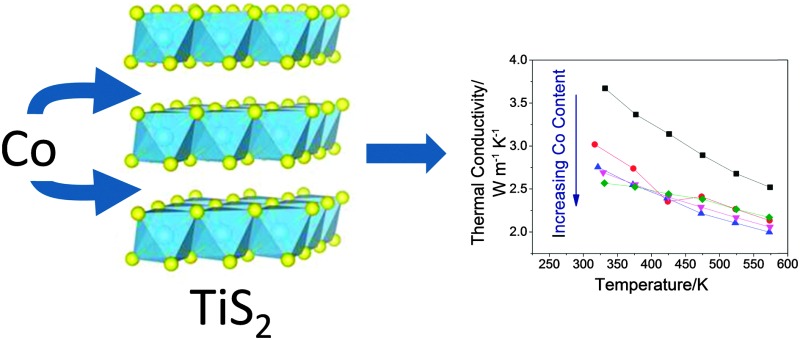
The thermoelectric figure of merit of TiS_2_ is increased by 25% through the intercalation of low levels of cobalt due to an increased electrical conductivity, arising from charge transfer, and a reduced thermal conductivity resulting from disorder.

## Introduction

**Fig. 1:**
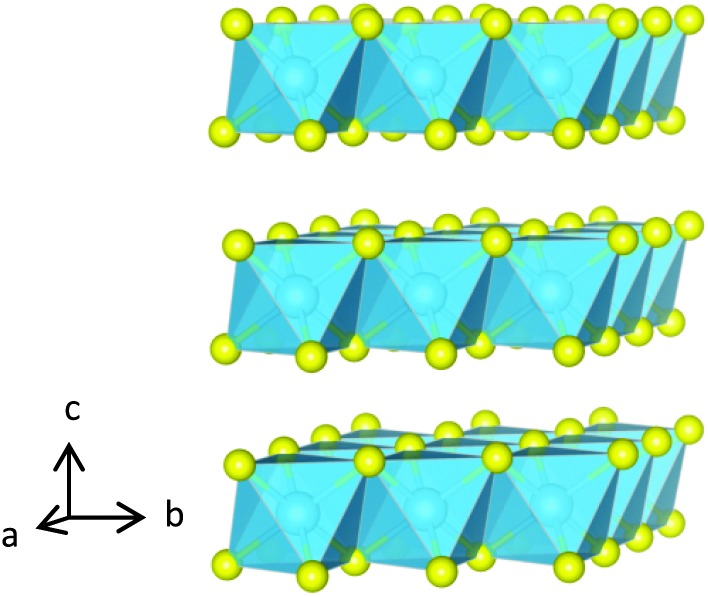
The structure of 1T-TiS_2_ comprised of Ti-centered octahedra, shown in blue, with sulphur anions in yellow.

**Fig. 2:**
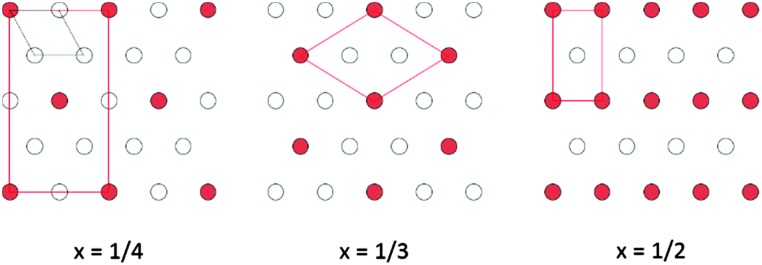
Possible 2-dimensional superstructures of A_*x*_MS_2_ for incomplete (*x* < 1) occupation of octahedral sites in the van der Waals' gap. The dashed line shows the primitive hexagonal cell of the pristine (*x* = 0) phase, whilst the red line outlines the supercells.

TiS_2_ is one of a number of transition-metal dichalcogenides whose structures consist of two-dimensional metal–chalcogen blocks, separated by a van der Waals' gap. TiS_2_ adopts the 1T structure (space group *P*3*m*1) consisting of two-dimensional slabs of edge-linked TiS_6_ octahedra (Fig. 1). The van der Waals' gap between successive TiS_2_ blocks contains a network of vacant octahedral and tetrahedral sites. The layered structure of TiS_2_ permits the ready intercalation of a wide range of guest species, including monatomic cations,^1^ molecular ions^2,3^ and organic molecules.^4^ Incomplete occupation of the network of octahedral sites in the van der Waals' gap of dichalcogenides by monatomic guest species may give rise to a variety of two-dimensional superstructures (Fig. 2) in phases of general formula A_*x*_MS_2_ (*x* < 1.0). Furthermore, the incorporation of a guest species, A, is accompanied by the transfer of electronic charge from guest to host. This provides a means of tuning the electron-transport properties. The presence of guest ions may also induce changes in the lattice component of the thermal conductivity. In addition to the important insights into the complex interplay of composition, structure and properties that such phases provide, the capacity to control the physical properties of a layered disulphide through intercalation offers considerable attractions in the design of new thermoelectric materials for energy harvesting.

As an all solid-state technology, thermoelectric energy harvesting offers advantages in terms of reliability, providing issues of cost and efficiency can be resolved. The efficiency of a device is determined by the transport properties of the materials of which it is comprised, embodied in a figure of merit *ZT* = *S* ^2^ *σT*/*κ*, where *S*, *σ* and *κ* are the Seebeck coefficient, electrical and thermal conductivities respectively.^5^ Doped-derivatives of Bi_2_Te_3_ that form the basis of commercial devices, exhibit a figure of merit, *ZT*, that approaches unity at *ca.* 380 K.^6^ However, in addition to issues over the toxicity of tellurium, the relative scarcity of this element limits the opportunities for wide-scale implementation of thermoelectric technology. This has motivated the search for materials with technologically-viable thermoelectric performance that contain earth-abundant elements. It has been reported^7^ that the value of the thermoelectric power factor of TiS_2_ single crystals (*S* ^2^ *σ* = 3.71 mW m^–1^ K^–2^ at 300 K within the *ab* plane) is comparable with that of Bi_2_Te_3_, whereas the thermal conductivity is significantly higher, thereby reducing the figure of merit. The beneficial effects for thermoelectric performance of the low dimensionality of TiS_2_ coupled with the capacity to tune transport properties by intercalation has led to considerable interest in the layered disulphide, TiS_2_, as a candidate thermoelectric material.^4,8–11^

In this work, we have investigated the effects of cobalt incorporation on the electronic, magnetic and thermal properties of TiS_2_. The results demonstrate that there is a balance to be struck between the benefits of electron transfer in increasing the electrical conductivity and the deleterious impact on the Seebeck coefficient of the increase in charge-carrier density on intercalation. We show that the most promising thermoelectric properties are realised at relatively low levels of cobalt incorporation, where cobalt cations are disordered over the available inter-layer octahedral sites. The figure of merit at these levels of cobalt incorporation is *ca.* 25% higher than that of the binary TiS_2_, reaching values of *ZT* = 0.30 at 573 K. Such materials are therefore of interest for energy recovery from low-grade waste heat at temperatures up to 600 K, for which, despite the spectacular increases in *ZT* achieved at high temperatures, there remains a dearth of suitable low-cost materials.

## Experimental

TiS_2_ was synthesised from the elements Ti (Alfa Aesar, 325 mesh, 99.99%) and S (Sigma Aldrich, flakes, 99.99%). The sulphur flakes were dried at room temperature under vacuum before use. Stoichiometric amounts of the elements were ground together before being placed in a fused silica ampoule. This was evacuated to 10^–4^ Torr before sealing. The sealed ampoule was placed in a furnace and heated at 350 °C for 4 h and 650 °C for 12 h, using a heating and cooling rate of 1 °C min^–1^. Samples of Co_*x*_TiS_2_ (*x* = 0.02; 0.04; 0.06; 0.08; 0.1; 0.15; 0.2; 1/4; 0.3; 1/3; 0.4; 1/2; 2/3; 3/4) were prepared by grinding appropriate quantities of TiS_2_ and Co (Alfa Aesar, 99.99%) prior to heating in an evacuated, sealed fused-silica ampoule. The mixture was heated at 650 °C for 2 successive periods of 48 h (heating/cooling rate 1 °C min^–1^) with an intermediate regrinding. For both the TiS_2_ synthesis and the cobalt intercalation step it is critically important to use a slow cooling rate to allow the sulphur to reintegrate into the powder without condensing on the wall of the tube.

Powder X-ray diffraction data for all samples were collected using a Bruker D8 Advance Powder X-ray diffractometer, operating with Ge monochromated CuKα_1_ radiation (*λ* = 1.54046 Å) and fitted with a LynxEye detector. Data were collected over the angular range 5 ≤ 2*θ*/° ≤ 120 for a period of 6 hours. Time-of-flight powder neutron diffraction data for selected samples of Co_*x*_TiS_2_ (*x* = 0.2, 1/4, 1/3, 1/2 and 2/3) were collected using the GEM diffractometer at the ISIS Facility, Rutherford Appleton Laboratory, UK. Data were collected for samples contained in thin-walled vanadium cans at room temperature and at temperatures to 2 K, using a vanadium-tailed cryostat. Initial data manipulation and reduction, including the subtraction of the empty cryostat signal for low-temperature measurements was carried out using the Mantid software package^12^ Rietveld analysis of both powder X-ray and neutron diffraction data was carried out using the GSAS package^13^ and residual maps plotted with DRAWxtl.^14^

Magnetic susceptibility data for Co_*x*_TiS_2_ (*x* = 0.2, 1/4, 1/3, 1/2 and 2/3) were obtained using a Quantum Design MPMS XL magnetometer. Data were collected over the temperature range 2.5 ≤ *T*/K ≤ 298 both after zero-field cooling (ZFC) and after cooling in the measuring field of 0.01 T (FC). Samples of Co_*x*_TiS_2_ for transport property measurements were consolidated using a hot press constructed in house. Powders were loaded into a graphite mould and pressed using tungsten carbide dies at 100 MPa for 30 min at 903 K under a N_2_ atmosphere. Prior to consolidation, ball-milling was used to homogenise and reduce the particle size of the powders. Ball milling was carried out in a 25 mL stainless-steel jar with 6 mm stainless-steel balls, under an argon atmosphere in a Retsch Planetary Ball Mill PM100 at 350 rpm for 1 hour with 5 min interval steps, with a change of direction. The weight ratio of powder to balls was 3 : 10. Consolidation produced high density compacts with ≥95% of the crystallographic value for low cobalt contents (*x* ≤ 0.08), 92% for *x* = 0.1 and *ca.* 80% for *x* ≥ 0.2 The densities of the consolidated pellets were measured using an Archimedes balance ADAM PW184. The consolidated pellets were cut using a MTI SYJ-150 digital diamond saw and carefully polished using a MTI EQ-Unipol-300 grinder/polisher to obtain parallel faces, with less than 0.05 mm variation in width.

**Fig. 3:**
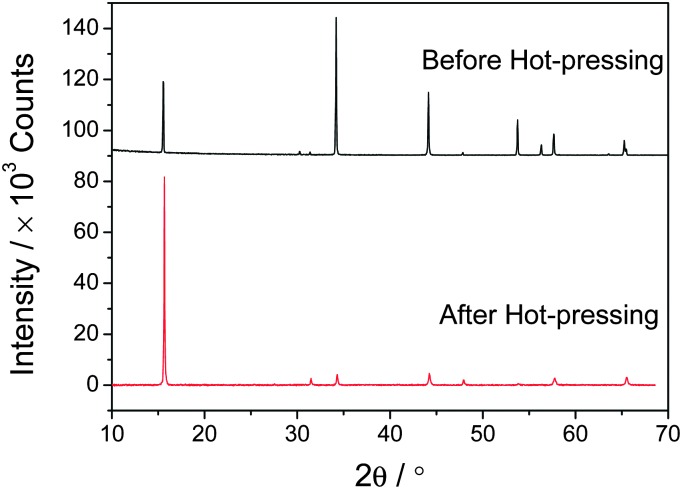
X-ray diffraction patterns of TiS_2_ powder prior to hot pressing and the TiS_2_ pellet following hot-pressing, illustrating the preferred orientation in the consolidated sample.

Powder X-ray diffraction data provide evidence of significant preferred orientation in the consolidated materials (Fig. 3). For this reason, physical property measurements were conducted both parallel to the pressing direction, hereafter referred to as cross plane, and perpendicular to the pressing direction, referred to as in plane. The degree of preferred orientation in consolidated materials becomes less marked with increasing values of *x*. This is illustrated by the ratio, *R*, of the intensities of the (001) against the strongest reflection (101) (ESI,† Fig. S1). *R*, which exceeds 30 for TiS_2_, is reduced to *ca.* 4 for Co_0.5_TiS_2_, where powder pattern simulations indicate ratios of 0.57 and 0.37 respectively in the absence of preferred orientation. Cross-plane thermal diffusivity measurements (303 ≤ *T*/K ≤ 573) were carried out using a Netzsch LFA 447 NanoFlash instrument on polished disks of *ca.* 2 mm width and *ca.* 12.7 mm diameter, whilst in-plane measurements (323 ≤ *T*/K ≤ 573) were performed using a Netzsch LFA 457 MicroFlash instrument in N_2_ atmosphere on plates of *ca.* 6 × 6 × 2 mm^3^ cut from pellets 8–10 mm in height. The thermal conductivity was calculated using the Dulong–Petit heat capacity (*C*_p_ = 0.668 J K^–1^ g^–1^ for TiS_2_; *C*_p_ = 0.656 J K^–1^ g^–1^ for Co_0.10_TiS_2_). In-plane and cross-plane electrical property measurements were made on ingots of *ca.* 2 × 2 × 8–10 mm^3^, cut from the large pellets.

The temperature dependence of both the electrical resistivity (*ρ*) and Seebeck coefficient (*S*) was measured simultaneously over the temperature range 303 ≤ *T*/K ≤ 573 using a Linseis LSR-3 instrument with samples contained under a partial pressure of He. A temperature gradient of 30 K was applied across the ends of the ingot for Seebeck coefficient measurements and a 50 mA current was passed for resistivity measurements.

Simultaneous TGA and DSC analysis of Co_*x*_TiS_2_ (*x* = 0, 0.08, 0.25, 0.33) was carried out using a TA instruments SDT Q600 thermal analyser. The analysis was conducted on *ca.* 25 mg of finely ground powder over the temperature range 303 ≤ *T*/K ≤ 1073, heating at a rate of 5 K min^–1^. Measurements were made under flows of both nitrogen and air.

## Results and discussion

All investigated compositions of Co_*x*_TiS_2_ were confirmed to be single phases by powder X-ray diffraction. Powder X-ray diffraction data for Co_*x*_TiS_2_ phases with *x* ≤ 0.15 are well described by a structural model in which cobalt atoms are completely disordered over octahedral sites in the inter-layer space. Observed, calculated and difference profiles are presented as ESI† (Fig. S2). The introduction of inter-layer cobalt cations causes a significant decrease in the *c*-lattice parameter relative to that of TiS_2_; changes in the in-plane (*a*) lattice parameter being less marked (Table 1; ESI,† Fig. S3). This behaviour is consistent with previous reports for cobalt intercalation^15^ that differs from most guest-TiS_2_ systems where increasing intercalation levels led to an increase in the *c* lattice parameter. Recent examples of such systems include Cu_*x*_TiS_2_,^10^ Ag_*x*_TiS_2_^8^ and misfit phases, (SnS)_1.2_(TiS_2_)_2_.^16^

**Table 1:** Refined structural parameters from Rietveld analysis of powder X-ray diffraction data for Co_*x*_TiS_2_ with *x* ≤ 0.15. (space group *P*3*m*1^*a*^)

		*x* in Co_*x*_TiS_2_						
		0	0.02	0.04	0.06	0.08	0.1	0.15
	*a*/Å	3.40676(5)	3.40527(6)	3.40442(5)	3.40321(5)	3.40311(8)	3.40302(4)	3.3997(2)
	*c*/Å	5.69716(8)	5.6841(1)	5.67862(9)	5.6687(1)	5.6616(2)	5.6550(1)	5.6341(3)
Ti	*B*/Å^3^	0.44(5)	0.45(6)	0.71(6)	0.74(6)	0.34(7)	0.36(8)	0.12(12)
S	*z*	0.2487(4)	0.2501(4)	0.2481(4)	0.2497(4)	0.2514(5)	0.2526(5)	0.2521(9)
	*B*/Å^3^	0.31(4)	0.27(5)	0.50(4)	0.42(5)	0.19(5)	0.07(5)	0.33(9)
Co	*B*/Å^3^	—	0.45(6)	0.71(6)	0.74(6)	0.34(7)	0.36(8)	0.12(12)
	*R*_wp_/%	8.4	8.5	7.5	7.2	6.9	6.7	6.8
	*χ* ^2^	1.33	1.33	1.28	1.31	1.17	1.45	1.42

^*a*^Ti on 1(a), (0, 0, 0); S on 2(d), (1/3, 2/3, *z*); Co on 1(b) (0, 0, 1/2). SOF(Co) constrained at nominal composition.

Whilst powder X-ray diffraction data provide evidence of the formation of superstructures at higher cobalt contents, their detailed structural characterisation using Cu-K_α_ radiation is hampered by the increased background signal arising from X-ray fluorescence associated with cobalt. This is particularly marked at low scattering angles (2*θ* ≤ 20°), where the relatively weak long *d*-space reflections characteristic of superstructure formation are expected to occur. Therefore, structural characterization of Co_*x*_TiS_2_ materials with *x* ≥ 0.2, was performed using powder neutron diffraction. Powder neutron diffraction data from the 2*θ* = 156°, 90°, 63° and 35° banks of the GEM diffractometer were summed, normalised and used in a multibank Rietveld refinement.

**Fig. 4:**
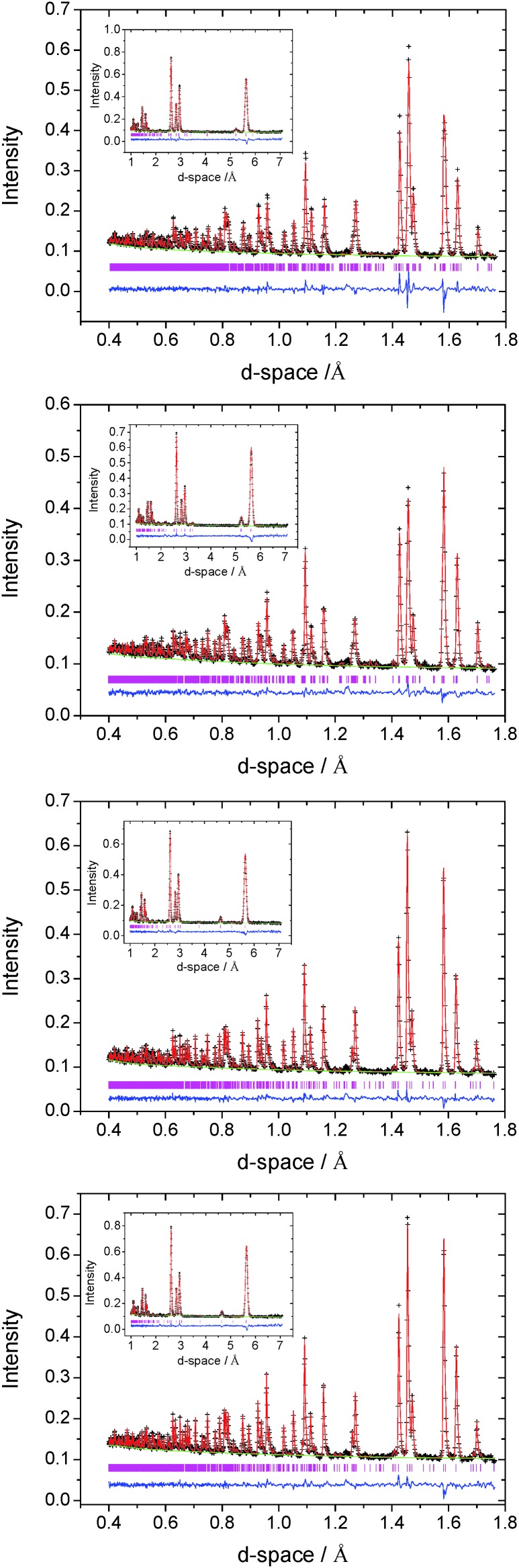
Observed (crosses), calculated (full line) and difference (lower full line) profiles for Co_*x*_TiS_2_ with *x* = 0.25; 0.5; 0.33; 0.4 from top to bottom, from Rietveld refinement against powder neutron diffraction data from the 2*θ* = 156° detector bank. Data at longer *d*-space (2*θ* = 35°) are shown as an inset and reflection positions are marked.

Powder neutron diffraction data for Co_0.25_TiS_2_ can be indexed on a monoclinic unit cell with lattice parameters related to those of the primitive hexagonal sub-cell by *a* ≈ 2√3*a*_p_, *b* ≈ 2*a*_p_, *c* ≈ 2*c*_p_. The data are well described (Fig. 4) by a model based on the monoclinic M_5_S_8_ (*x* = 1/4) structure, for which the two-dimensional cation-vacancy ordering scheme is shown schematically in Fig. 2. The powder neutron diffraction data for Co_0.5_TiS_2_ are also indexable on a monoclinic unit cell, but with lattice parameters related to the underlying hexagonal sub-cell through *a* ≈ √3*a*_p_, *b* ≈ *a*_p_, *c* ≈ 2*c*_p_. Data (Fig. 4) are well fitted by a structural model based on the M_3_S_4_ (*x* = 1/2) superstructure of Fig. 2, in which 50% occupancy of interlayer sites results in chains of Co-centered octahedra within the van der Waals' gap of TiS_2_. Refined atomic parameters for the two monoclinic ordered-defect structures are presented in Table 2.

**Table 2:** Refined structural parameters of the ordered defect structures of Co_0.25_TiS_2_ (space group *F*2/*m*)^*a*^ and Co_0.5_TiS_2_ (space group *I*2/*m*)^*b*^

		*x* in Co_*x*_TiS_2_	
		0.25	0.50
	*a*/Å	11.7708(4)	5.8943(2)
	*b*/Å	6.8038(2)	3.40429(9)
	*c*/Å	11.2444(2)	11.2350(2)
	*β*/°	90.31(2)	90.278(2)
Ti(1)	*x*	0.5004(8)	0.0100(5)
	*z*	0.2445(4)	0.2555(2)
	*B*/Å^3^	0.47(2)	0.37(2)
Ti(2)	*y*	0.251(2)	—
	*B*/Å^3^	0.47(2)	
S(1)	*x*	0.1674(8)	0.3301(5)
	*z*	0.1248(5)	0.3762(2)
	*B*/Å^3^	0.34(2)	0.22(2)
S(2)	*x*	0.1640(7)	0.3344(5)
	*z*	0.1157(4)	0.8822(2)
	*B*/Å^3^	0.34(2)	0.22(2)
S(3)	*x*	0.4173(4)	—
	*y*	0.2491(9)	—
	*z*	0.1245(3)	—
	*B*/Å^3^	0.34(2)	—
Co	*B*/Å^3^	1.11(7)	0.74(3)
	*R*_wp_/%	3.1	2.6
	*χ* ^2^	2.13	2.05

^*a*^Co(1) on 4(a), (0, 0, 0); Ti(1), S(1), S(2) on 8(i), (*x*, 0, *z*); Ti(2) on (1/4, *y*, 1/4); S(3) on 16(j), (*x*, *y*, *z*).

^*b*^Ti(1), S(1), S(2) on 4(i), (*x*, 0, *z*); Co(1) on 2(a), (0, 0, 0)

At *x* = 0.33 and 0.40, a M_2_S_3_-type structure (space group *P*31*c*) is adopted with a unit cell related to that of the primitive hexagonal cell by *a* ≈ √3*a*_p_, *b* ≈ √3*a*_p_, *c* ≈ 2*c*_p_. Refined atomic parameters are presented in Table 3. For *x* = 0.33, refinement of the site occupancy factor of Co(1) from its ideal value of unity, was required to produce a satisfactory fit. Allowing for the possibility of cobalt being located at either of the other two octahedral positions, Co(2) and Co(3), led to a non-zero site occupancy factor for Co(2) (0, 0, 0.25) only. The structure of Co_0.33_TiS_2_ therefore closely approaches the ideal M_2_S_3_-type with more than 85% of cobalt at the expected site and a refined stoichiometry of Co_0.31(2)_TiS_2_. The structure of Co_0.4_TiS_2_ is similar, with the additional cobalt cations being distributed over the two octahedral positions, Co(2) and Co(3), both normally vacant in M_2_S_3_. Removing the stoichiometry constraint on site occupancy factors during refinement does not lead to significant deviations from their constrained values, nor does it produce any apparent improvement in the quality of refinement. Final observed, calculated and difference profiles for neutron diffraction data of Co_0.33_TiS_2_ and Co_0.40_TiS_2_ are presented in Fig. 4.

**Table 3:** Refined parameters from Rietveld analysis of powder neutron diffraction data for Co_*x*_TiS_2_, *x* = 0.33, 0.40 (space group *P*31*c*)^*a*^

		*x* in Co_*x*_TiS_2_	
		0.33	0.40
	*a*/Å	5.88111(4)	5.88235(4)
	*c*/Å	11.2573(2)	11.2563(2)
Ti(1)	*B*/Å^3^	0.40(2)	0.41(2)
Ti(2)	*z*	–0.0047(2)	–0.0048(3)
	*B*/Å^3^	0.40(2)	0.41(2)
S	*x*	0.333(1)	0.333(1)
	*y*	–0.0006(4)	0.0001(4)
	*z*	0.37192(6)	0.37142(6)
	*B*/Å^3^	0.33(2)	0.29(2)
Co(1)	SOF	0.852(9)	1.0
	*B*/Å^3^	0.41(5)	0.82(4)
Co(2)	SOF	0.09(1)	0.151(9)
	*B*/Å^3^	0.41(5)	0.82(4)
Co(3)	SOF	—	0.049(9)
	*B*/Å^3^	—	0.82(4)
	*R*_wp_/%	2.7	2.7
	*χ* ^2^	1.56	1.76

^*a*^Ti(1) on 2(d), (0, 0, 0); Ti(2) on 4(f), (1/3, 2/3, *z*); S on 12(i), (*x*, *y*, *z*); Co(1) on 2(c), (1/3, 2/3, 0.25); Co(2) on 2(a), (0, 0, 0.25); Co(3) on 2(b), (2/3, 1/3, 0.25).

**Fig. 5:**
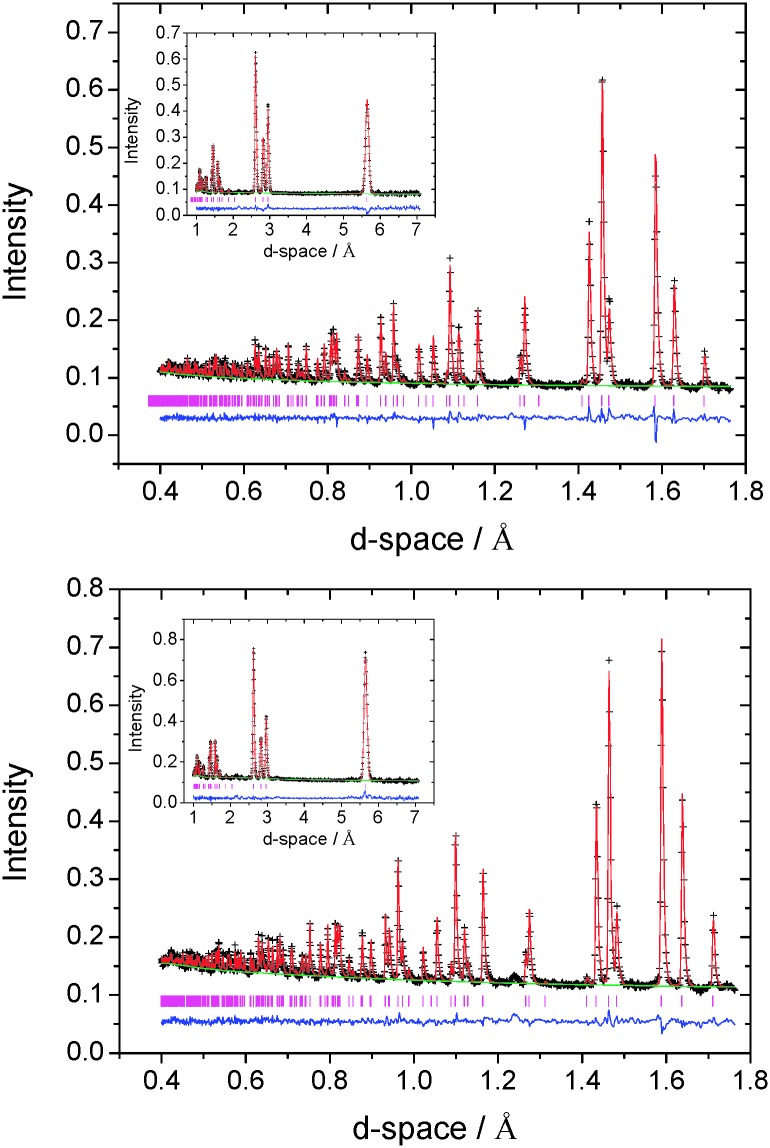
Observed (crosses), calculated (full line) and difference (lower full line) profiles for Co_0.2_TiS_2_ (top) and Co_0.67_TiS_2_ (bottom) from Rietveld refinement against powder neutron diffraction data from the 2*θ* = 156° detector bank. Data at longer *d*-space (2*θ* = 35°) are shown as an inset and reflection positions are marked.

**Fig. 6:**
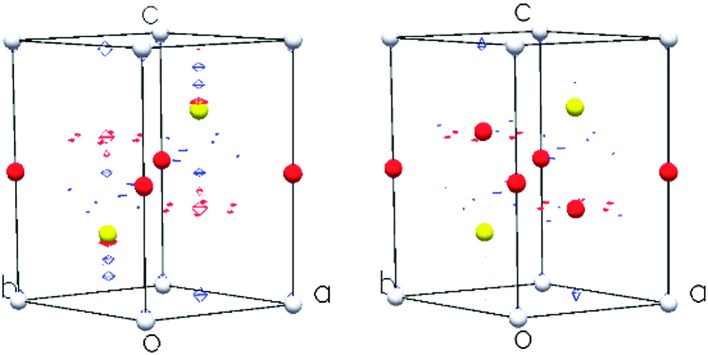
Difference Fourier maps of Co_0.67_TiS_2_ without (left) and with (right) cobalt in tetrahedral positions. Red and blue contours correspond to positive and negative regions with identical amplitudes.

**Fig. 7:**
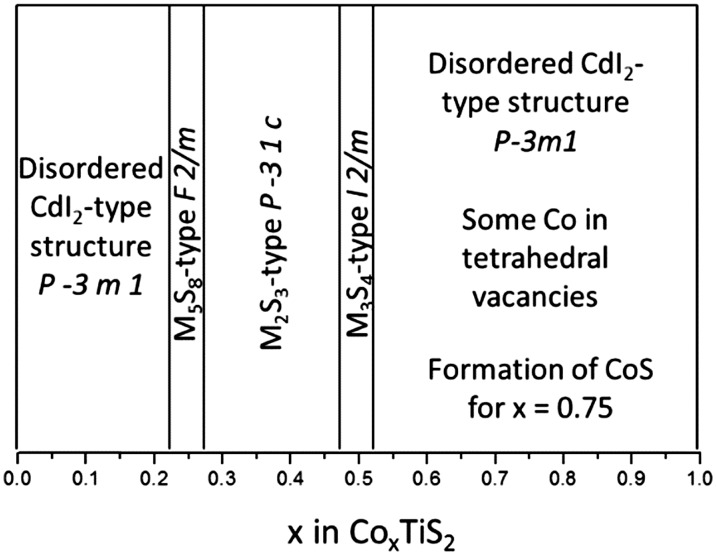
Structural arrangements of Co_*x*_TiS_2_ as a function of the cobalt intercalation level, *x*, established from XRD and neutron diffraction analysis.

By contrast, at the extremes of composition investigated using neutron diffraction (*x* = 0.2, 0.67), the data (Fig. 5) provide no evidence for ordering of cobalt ions over octahedral sites (Table 4). The data for Co_0.2_TiS_2_ are consistent with a structure (space group *P*3*m*1), analogous to that determined by X-ray methods for Co_*x*_TiS_2_ (0.02 ≤ *x* ≤ 0.15), in which there is a disordered distribution of cobalt ions over the inter-layer octahedral sites. However, a disordered distribution of cobalt ions over inter-layer octahedral sites does not adequately describe the powder neutron diffraction data for Co_0.67_TiS_2_. In addition to higher than expected values of the weighted residual, there is a large discrepancy between the crystallographically determined composition (Co_0.43(1)_TiS_2_) and the nominal stoichiometry. The difference Fourier map (|*F*_obs_ – *F*_calc_|), (Fig. 6), indicates residual nuclear density at positions corresponding to inter-layer tetrahedral sites. Introduction of cobalt into these positions improves the quality of the refinement markedly, and leads to a refined composition Co_0.624(8)_TiS_2_, closer to the nominal stoichiometry, albeit with a slight remaining Co deficiency. To the best of our knowledge this represents a unique example of an A_*x*_MS_2_ phase in which both octahedral and tetrahedral inter-layer sites are simultaneously occupied. The evolution of the structure of Co_*x*_TiS_2_ phases with composition, determined from the combination of X-ray and neutron diffraction, is shown schematically in Fig. 7.

**Table 4:** Refined parameters from Rietveld analysis of powder neutron diffraction data for Co_*x*_TiS_2_, *x* = 0.2, 0.67 (space group *P*3*m*1)^*a*^

		*x* in Co_*x*_TiS_2_	
		0.2	0.67
	*a*/Å	3.40037(3)	3.42065(3)
	*c*/Å	5.63223(7)	5.64181(8)
Ti	*B*/Å^3^	0.56(2)	0.73(2)
S	*z*	0.2539(2)	0.2591(2)
	*B*/Å^3^	0.43(2)	0.53(2)
Co(1)	SOF	0.2(—)	0.484(4)
	*B*/Å^3^	0.81(8)	0.69(5)
Co(2)	*z*	—	0.354(3)
	SOF	—	0.070(2)
	*B*/Å^3^	—	0.69(5)
	*R*_wp_/%	2.9	2.7
	*χ* ^2^	1.70	1.95

^*a*^Ti on 1(a), (0, 0, 0); S on 2(d), (1/3, 2/3, *z*); Co(1) on 1(b), (0, 0, 1/2); Co(2) on 2(d), (2/3, 1/3, *z*).

Kawasaki *et al.* ^17^ have recently re-investigated the structural effect of intercalation in Co_*x*_TiS_2_ using single crystal X-ray diffraction. They described the structure of materials with compositions corresponding to *x* = 0.26, 0.43 and 0.57 in the trigonal space groups *P*3*m*1 (*x* = 0.26, 0.57) and *P*31*c* (*x* = 0.43) with unit cells given by 2*a*_p_ × 2*a*_p_ × 2*c*_p_ and √3*a*_p_ × √3*a*_p_ × 2*c*_p_ respectively. However, in these models, intercalated cations show a high degree of disorder and those described in *P*3*m*1 involve an alternation of near fully ordered layers with almost completely disordered layers that is unexpected on energetic grounds.^18^ This contrasts with the conclusions drawn from powder neutron diffraction which shows an evolution of vacancy ordering in every other layer with increasing cobalt content that encompasses monoclinic M_5_S_8_, trigonal M_2_S_3_ and monoclinic M_3_S_4_ structures, some of which are stabilised over a range of compositions, in addition to disordered structures observed at the compositional extremes. Support for the conclusions drawn here is provided by previous studies^19–21^ which suggest monoclinic distortions in phases with *x* = 0.25 and 0.5 for Co_*x*_TiS_2_.

**Fig. 8:**
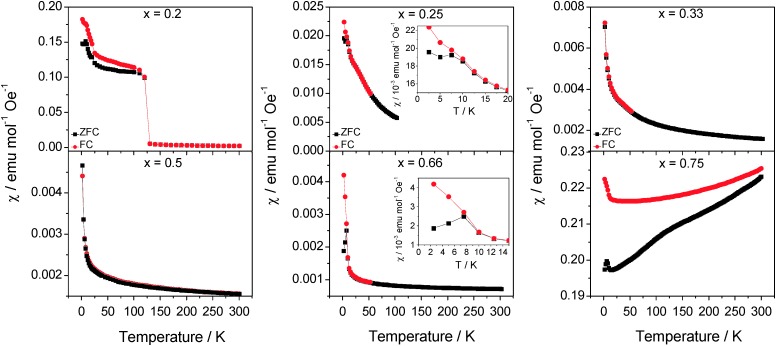
Zero-field cooled (black squares) and field-cooled (red circles) molar magnetic susceptibility of Co_*x*_TiS_2_ (*x* = 0.2; 0.25; 0.33; 0.5; 0.66 and 0.75). Insets show details in the region of anomalies in *χ*_mol_(*T*).

Magnetic susceptibility data (Fig. 8) provide further insights into the electronic states of these phases. The susceptibility data for Co_0.20_TiS_2_ clearly reveal a ferromagnetic transition at *T*_C_ = 130 K, although no magnetic scattering was detectable in powder neutron diffraction data collected at 2 K. This may be a consequence of the small ordered moment arising from the 20% occupancy of the Co(1) site. Inoue *et al.* ^22^ have observed a similar transition in AC susceptibility data for single crystals of Co_*x*_TiS_2_ (0.075 ≤ *x* ≤ 0.33). In the present work no magnetic ordering transitions were observed for *x* = 0.25 and *x* = 0.33, which may be attributable to differences in the homogeneity of the single crystal and powder samples. However, it is possible that these previous studies and the present Co_0.2_TiS_2_ sample contain trace amounts of CoS_2_, which is a ferromagnet with a Curie temperature reported between 110 K and 130 K.^23–25^ The magnetic susceptibility decreases with increasing cobalt content up to *x* = 0.66, consistent with the work of Danot *et al.* ^21^ Data in the paramagnetic regions are well described by a modified Curie–Weiss law (*χ* = *χ*_0_ + *C*/*T* – *θ*) incorporating a temperature independent term. At low cobalt contents, the effective magnetic moments (Table 5) are in reasonable agreement with the spin-only values for low-spin octahedral Co^2+^ (*μ*_eff_ = 1.73). However, as the cobalt content increases above *x* = 0.33, the moment deviates significantly from spin-only behaviour. This may be attributed to an increasing degree of electron delocalization as the concentration of cobalt in the vacancy layer increases. This observation supports the conclusion previously drawn by Inoue *et al.* ^1,22^ on the formation of a (t_2g_)^6^(e_g_)^1^ low-spin band near the Ti 3d band. The negative Weiss constant indicates that in all phases for *x* ≤ 0.66, the dominant magnetic exchange interactions are antiferromagnetic in origin with a weak ferromagnetic component observed for Co_0.25_TiS_2_ and Co_0.66_TiS_2_. Antiferromagnetic correlations may also serve to reduce the effective magnetic moment as evidenced by the decrease in the quantity (8*χT*/*n*)^1/2^ with decreasing temperature (ESI,† Fig. S4). At a composition Co_0.75_TiS_2_ very different behaviour is observed in the magnetic susceptibility data. The data appear to indicate a magnetic ordering transition that occurs above 300 K – this may be estimated as 350–400 K (ESI,† Fig. S5). This may be associated with the presence of trace amounts of CoS (*T*_N_ = 358 K),^24^ suggesting that the limit of the single-phase region of Co_*x*_TiS_2_ lies in the range 0.66 ≤ *x* ≤ 0.75.

**Table 5:** Derived magnetic parameters obtained from the fit of a modified Curie–Weiss law to magnetic susceptibility data over the temperature range specified

*x*	Temp. range (K)	*χ*_0_ (emu mol^–1^)	*θ* (K)	*μ*_eff_ per Co
0.2	150–300	—	–26(3)	2.36(7)
0.25	40–100	3.91(8) × 10^–4^	–13.5(5)	2.24(3)
0.33	40–300	1.100(7) × 10^–3^	–36(1)	1.15(4)
0.5	55–215	1.430(6) × 10^–3^	–45(2)	0.63(1)
0.67	25–220	6.70(2) × 10^–4^	–17(2)	0.37(3)

TGA/DSC analysis of Co_*x*_TiS_2_ (*x* = 0; 0.08; 0.25 and 0.33), performed under both inert (N_2_) and oxidising (air) atmospheres, are provided as ESI† (Fig. S6). All four samples produce an exothermic peak when heated in air, corresponding to rapid oxidation. Intercalation of cobalt shifts the exothermic peak to higher temperature; from 615 K for TiS_2_ to 713 K for Co_0.33_TiS_2_. Powder X-ray diffraction (ESI,† Fig. S7 and S8) identifies the oxidation products as being anatase TiO_2_ for *x* = 0; anatase with evidence for the rutile form at *x* = 0.08; and a mixture of rutile and CoTiO_3_ for higher cobalt contents. In an inert atmosphere, no exotherms arising from oxidation are observed, however a slow oxidation due to the presence of trace amounts of oxygen occurs over the 160 min of the thermal analysis run, leading to formation of rutile and anatase TiO_2_ in addition to the Co_*x*_TiS_2_ phases. For TiS_2_ and Co_0.08_TiS_2_, weight loss associated with sulphur volatilisation occurs with an onset in the range 473 ≤ *T*/K ≤ 573, suggesting that a protective coating would be required for applications at temperatures above 573 K. To avoid degradation of the samples through sulphur volatilization, all transport measurements were therefore performed at temperatures below 573 K.

**Fig. 9:**
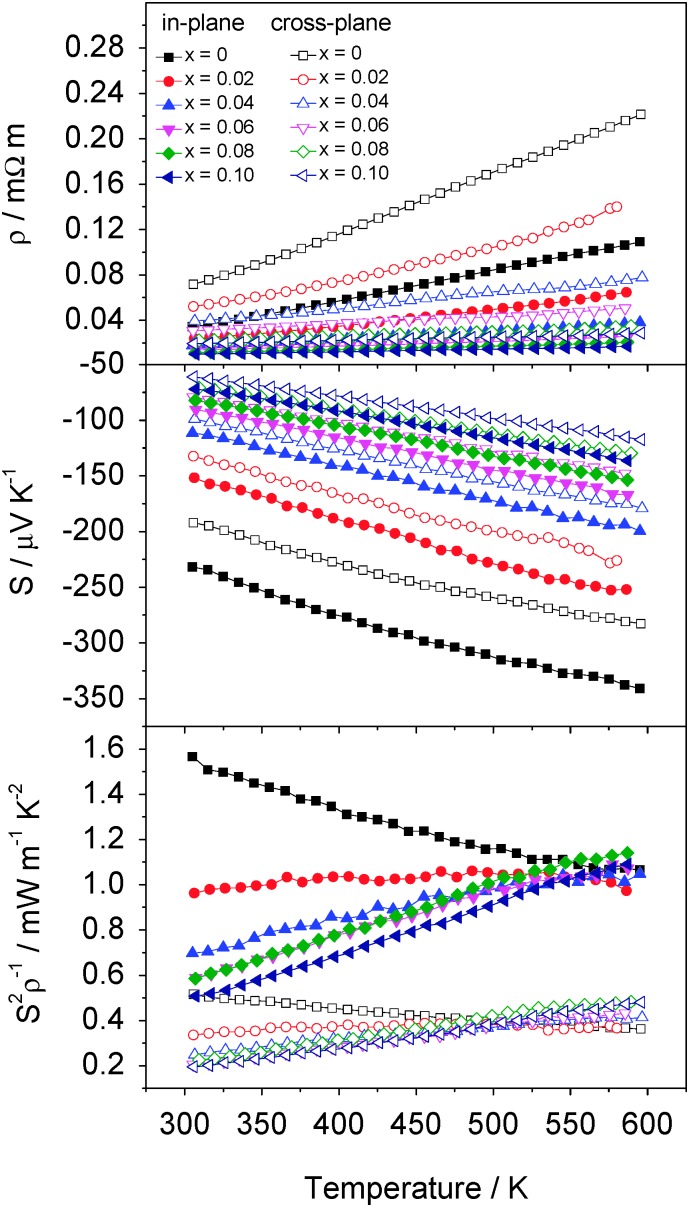
Temperature dependence of the electrical resistivity (*ρ*), Seebeck coefficient (*S*) and power factor (*S* ^2^ *ρ* ^–1^) of Co_*x*_TiS_2_ (0 ≤ *x* ≤ 0.10). Full symbols represent measurements made along the in-plane direction while the empty symbols correspond to cross-plane measurements.

The Seebeck coefficient (Fig. 9) determined here for polycrystalline TiS_2_ at 300 K is in good agreement with the measurements by Imai *et al.* ^7^ on single crystals: The value of *S* = –232 μV K^–1^ in the present study for the in-plane direction compares favourably that of *S* = –251 μV K^–1^ at 300 K within the *ab* plane in single crystals. Similarly, *ρ* = 0.034 mΩ m in the in-plane direction for the hot pressed sample is of the same order of magnitude to that determined in the *ab* plane for the single crystals (*ρ* = 0.017 mΩ m). Notably, the cross-plane resistivity is reduced by two orders of magnitude from that along the crystallographic *c* direction in the single crystal, indicating the less than perfect alignment of plate-like crystallites in the hot-pressed polycrystalline sample. The electrical properties of TiS_2_ have been the subject of some debate as discrepancies in physical properties determined by different groups led to uncertainty over whether 1T-TiS_2_ is a semi-metal^26,27^ or a semiconductor, with different values of direct or indirect band gap having been reported. However, it is now understood that the origin of the variations in physical properties lies in sulphur volatilisation at high temperatures. This results in titanium self-intercalation into the van der Waals' gap. The properties of the resulting phases formulated Ti_1+*γ*_S_2_, are sensitively dependent on the level of non-stoichiometry.^9,28,29^ In an effort to minimise the effects of sulphur volatilization and the resulting self-intercalation of titanium, controlled cooling was adopted in the present work and identical conditions were adopted for synthesis and consolidation of all samples The success of this approach is reflected in the Seebeck coefficient, the magnitude of which provides a sensitive probe of the degree of non-stoichiometry in the parent phase.^9^ Comparison between in-plane measurements reported here and those for Ti_1+*γ*_S_2_ that we have previously reported provides an estimate of the upper limit of non-stoichiometry of 1.5%, corresponding to a composition Ti_1.015_S_2_. This is also consistent with a *c* lattice parameter of <5.70 Å and a unit cell volume, *V* < 57.3 Å^3^, both of which suggest the deviation from stoichiometry lies in the range 1–1.5%.

Electrical resistivity and Seebeck coefficient measurements for Co_*x*_TiS_2_ over the range of composition 0 ≤ *x* ≤ 0.4 were initially carried out in plane (Fig. S9, ESI†). These measurements reveal that both the electrical resistivity and the absolute value of the Seebeck coefficient decrease markedly with increasing *x*. This can be attributed to the donation of electrons from the cobalt cation to the empty t_2g_-derived conduction band of TiS_2_, thereby increasing the charge carrier density at *E*_F_. This results in thermoelectric power factors below 0.3 mW m^–1^ K^–2^ for materials with compositions corresponding to *x* ≥ 0.2. By contrast, at lower cobalt contents, the power factor reaches values that are comparable to that of polycrystalline TiS_2_. Similarly, cross-plane thermal conductivity measurements for Co_*x*_TiS_2_ samples with *x* > 0.1, are significantly higher than those for materials with lower Co contents. This may be attributed in part to an increase in charge carrier concentration due to the transfer of electrons from Co to the TiS_2_ host. However, the electronic contribution to the thermal conductivity (*κ*_el_), (ESI,† Fig. S10) calculated from the Wiedemann–Franz law decreases again above *x* = 0.25 due to the increased electrical resistivity which may be associated with the lower level of densification of materials with higher cobalt levels. The overall thermal conductivity however remains high for these samples despite an increased porosity, (ESI,† Fig. S11) suggesting that the lattice contribution also increases with cobalt intercalation. For these reasons, the thermoelectric properties of samples with higher Co contents were not investigated in further detail and henceforth we focus on the thermoelectric behaviour of Co_*x*_TiS_2_ with *x* ≤ 0.10.

The electrical resistivity measured in the in-plane and in the cross-plane directions of Co_*x*_TiS_2_ (*x* ≤ 0.10) (Fig. 9) decreases with decreasing temperature: behaviour reminiscent of a metal. The Seebeck coefficient of Co_*x*_TiS_2_ materials is negative for all values of *x* with the absolute value increasing with *T*. This is consistent with the dominant charge carriers being electrons in all materials studied. Despite the apparent metal-like temperature dependence of the resistivity, values of the Seebeck coefficient are generally considerably higher than anticipated for a conventional metal, suggesting that the materials are better described as degenerate semiconductors. Both electrical resistivity and Seebeck coefficient are higher for cross-plane measurements compared to in-plane measurements from the same pellet. This anisotropy is attributed to the 2-dimensional layered structure and the preferential alignment of the crystallites during consolidation.

Whilst increasing cobalt intercalation reduces both the electrical resistivity and Seebeck coefficient, at low cobalt contents the gain from the former is sufficient at higher temperatures to compensate for the impact of the reduction in Seebeck coefficient. This produces a comparable thermoelectric power factor (*S* ^2^ *ρ* ^–1^) to that of the pristine phase (Fig. 9) at temperatures above 500 K for cross-plane and above 575 K for in-plane measurements. The power factor for Co_*x*_TiS_2_ (0 ≤ *x* ≤ 0.10) reaches *ca.* 1–1.1 mW m^–1^ K^–2^ over the 550 K to 595 K range in the in-plane direction, while the cross-plane electrical properties are significantly lowered by the preferred orientation and the anisotropy highlighted by the single-crystal properties with power factors ranging from 0.35 to 0.45 mW m^–1^ K^–2^ over the 500 K to 595 K range.

**Fig. 10:**
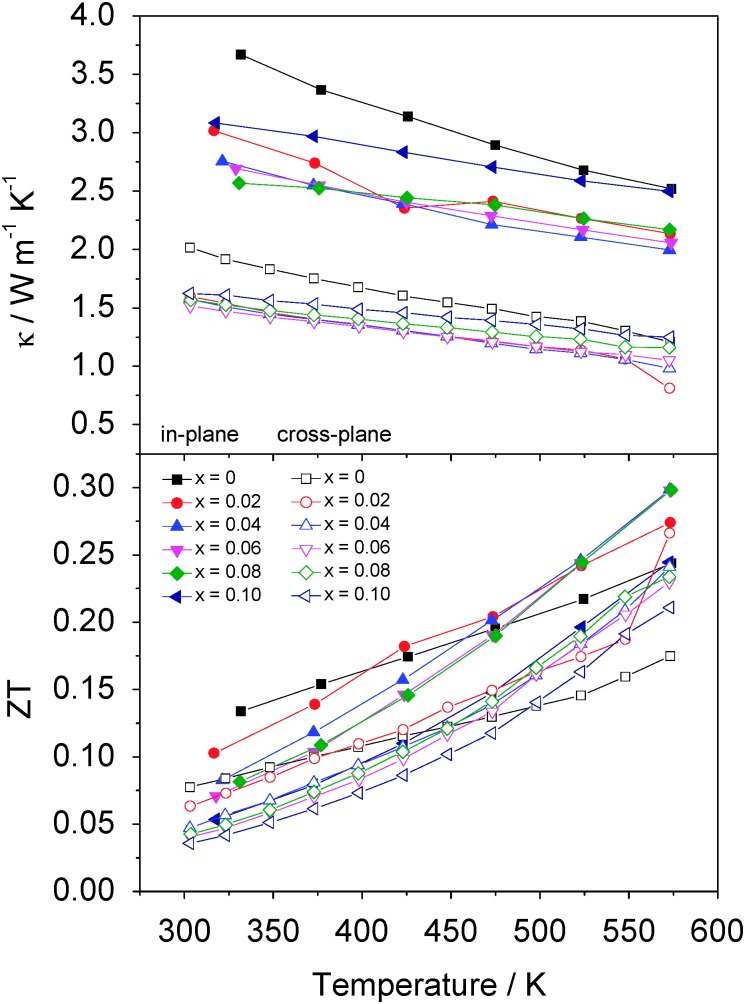
Temperature dependence of the thermal conductivity (top) and *ZT* (bottom) of Co_*x*_TiS_2_. (0 ≤ *x* ≤ 0.10). In-plane and cross plane data are indicated by solid and open symbols respectively.

In contrast with the behaviour of Co_*x*_TiS_2_ materials with high cobalt contents discussed above, significant reductions in thermal conductivity may be realised at compositions corresponding to *x* ≤ 0.08 (Fig. 10). Thermal conductivity data reveal that these reductions are achieved despite the increased charge carrier contribution (*κ*_el_), as a consequence of electrons donated from cobalt. Subtraction of this charge carrier contribution, estimated from the Wiedemann–Franz law (*L* = 2.44 × 10^–8^ W Ω K^–2^) reveals that the introduction of low levels of cobalt into the inter-layer space of TiS_2_ effects marked reductions in the lattice contribution to the thermal conductivity (*κ*_L_). This suggests that a disordered array of cobalt ions in the van der Waals' gap of the metal dichalcogenide structure provides an effective means of scattering heat carrying phonons. The effect is particularly marked along the in-plane direction with *κ* decreasing by 20% on going from the pristine phase to Co_0.04_TiS_2_.

**Fig. 11:**
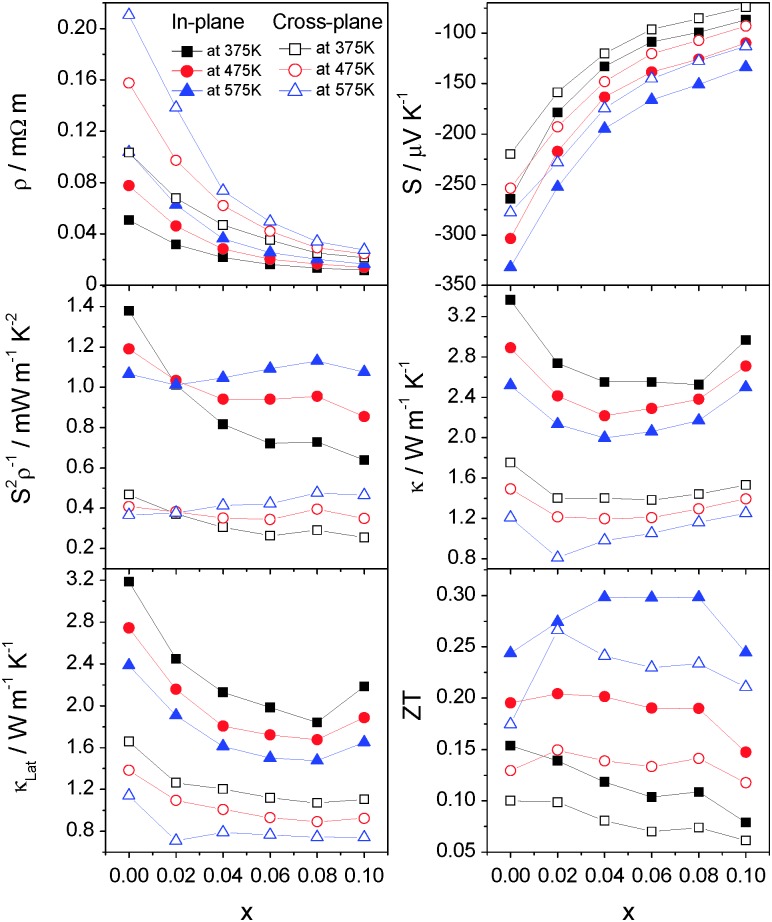
Compositional dependence of the in-plane (solid symbols) and cross-plane (open symbols) thermoelectric performance of Co_*x*_TiS_2_ (0 ≤ *x* ≤ 0.10), including electrical resistivity (top left), Seebeck coefficient (top right), power factor (middle left), thermal conductivity (middle right), electronic contribution to thermal conductivity (bottom left) and *ZT* (bottom right).

The compositional dependence of the in-plane and cross-plane electrical resistivity, Seebeck coefficient, power factor, lattice and total thermal conductivities and figure of merit for Co_*x*_TiS_2_ (*x* ≤ 0.1) at selected temperatures is shown in Fig. 11. For all compositions, the in-plane and cross-plane properties exhibit a similar compositional dependence. A rapid fall in electrical resistivity and the corresponding decrease of |*S*| with increasing cobalt content results in a change in the temperature dependence of the power factor at *x* = 0.02 from a negative to a positive gradient. The lattice contribution to the thermal conductivity also decreases rapidly with cobalt intercalation up to *x* = 0.08 with a slightly more pronounced effect in the in-plane than in the cross plane direction. Despite the rapid rise in the electronic contribution, the total thermal conductivity is reduced and a flat minimum is observed over the range 0.02 ≤ *x* ≤ 0.06. The maintenance of a reasonably high power factor on intercalation combined with a reduced thermal conductivity produces an enhancement of *ZT* over that of TiS_2_. Whilst all samples with low levels of intercalation (*x* ≤ 0.08) show an enhancement over the temperature range investigated, the best performance is achieved for materials with compositions in the range 0.04 ≤ *x* ≤ 0.08, that exhibit *ZT* = 0.3 at 573 K in the in-plane direction, a 25% increase over the maximum value of that for TiS_2_ at the same temperature. The effect of divalent cobalt intercalation is similar to that of monovalent copper^10,30^ and silver^8^ with intercalation simultaneously decreasing the electrical resistivity and thermal conductivity. The maximum *ZT* = 0.3 reported here for hot-pressed samples compares favourably with M_*x*_TiS_2_ (M = Cu, Ag, Ti) at 573 K prepared by SPS.

## Conclusions

Using a combination of powder X-ray and neutron diffraction, we have established a structural phase diagram of Co_*x*_TiS_2_ (0 ≤ *x* ≤ 0.75) which exhibits, with increasing cobalt content an evolution from a disordered phase through three superstructure types, monoclinic M_5_S_8_, trigonal M_2_S_3_ and monoclinic M_3_S_4_, to a second disordered region at the highest cobalt contents in which both octahedral and tetrahedral sites are occupied. The intercalation of cobalt causes marked changes to the electron-transport properties of TiS_2_. In particular, electron transfer from cobalt to the conduction band of the TiS_2_ host results in a systematic reduction in the electrical resistivity in well-densified samples. Whilst the lower resistivity is advantageous from a thermoelectric perspective, the accompanying fall in |*S*| leads to low power factors in phases with higher cobalt contents. Moreover, the thermal conductivity remains high for samples with high cobalt content despite the increased porosity of the samples and the artificially low electronic contribution. Conversely, at low levels of cobalt incorporation, (*x* ≤ 0.10), the gain in electrical conductivity is sufficiently high to offset the reduction in the absolute value of the Seebeck coefficient, leading to a power factor comparable with or higher than that of pristine TiS_2_ over the Co_*x*_TiS_2_ series (0.02 ≤ *x* ≤ 0.1) at temperatures above 500 K for cross-plane and above 550 K for in-plane measurements. The disordered arrangement of cobalt cations over inter-layer octahedral sites also leads to a reduction in lattice thermal conductivity, *κ*_L_, through increased phonon scattering. The decrease in *κ*_L_ ameliorates the impact of the increased charge carrier contribution, leading to a decrease in overall thermal conductivity. The changes induced by incorporation of low levels of cobalt increases the figure of merit by *ca.* 25% over that of TiS_2_, with *ZT* reaching a maximum *ZT* = 0.3 for Co_*x*_TiS_2_ (0.04 ≤ *x* ≤ 0.08) at 573 K. Whilst higher *ZT* values have been reported for n-type sulphides, this level of performance is achieved at considerably higher temperatures with *ZT* ≥ 0.4 at 950 K for Cu_4_Mo_6_S_8_^31^ and LaGd_1.02_S_3_,^32^ and *ZT* ≥ 1 for doped-Bi_2_S_3_ at 923 K.^33^ For the temperature range appropriate to energy recovery from low grade waste heat that is the focus of the work described here, the maximum *ZT* attained is similar to that of M_*x*_TiS_2_ (M = Cu, Ag)^8,10^ and TiS_2_-based superlattices^3,11^ prepared by Spark Plasma Sintering and to the hot-pressed Shandite-type phase Co_3_Sn_1.6_In_0.4_S_2_.^34^ The recent report of comparable performance in a p-type synthetic bornite-type sulphide^35^ suggests that with further development, sulphide-based thermoelectrics may offer an attractive alternative to Bi_2_Te_3_ for thermoelectric energy recovery from waste heat at low temperatures using earth-abundant elements.

## References

[cit1] Inoue M., Hughes H. P., Yoffe A. D. (1989). Adv. Phys..

[cit2] Wiegers G. A., Haange R. J. (1991). Eur. J. Solid State Inorg. Chem..

[cit3] Wan C., Wang Y., Wang N., Koumoto K. (2010). Materials.

[cit4] Wan C., Gu X., Dang F., Itoh T., Wang Y., Sasaki H., Kondo M., Koga K., Yabuki K., Snyder G. J., Yang R., Koumoto K. (2015). Nat. Mater..

[cit5] Rowe D. M., in Thermoelectrics Handbook: Macro to Nano, ed. D. M. Rowe, CRC Press, 2006, ch. 1, pp. 1.1–1.13.

[cit6] Snyder G. J., Toberer E. S. (2008). Nat. Mater..

[cit7] Imai H., Shimakawa Y., Kubo Y. (2001). Phys. Rev. B: Condens. Matter Mater. Phys..

[cit8] Barbier T., Lebedev O. I., Roddatis V., Breard Y., Maignan A., Guilmeau E. (2015). Dalton Trans..

[cit9] Beaumale M., Barbier T., Breard Y., Guelou G., Powell A. V., Vaqueiro P., Guilmeau E. (2014). Acta Mater..

[cit10] Guilmeau E., Breard Y., Maignan A. (2011). Appl. Phys. Lett..

[cit11] Guilmeau E., Maignan A., Wan C., Koumoto K. (2015). Phys. Chem. Chem. Phys..

[cit12] Arnold O., Bilheux J. C., Borreguero J. M., Buts A., Campbell S. I., Chapon L., Doucet M., Draper N., Ferraz Leal R., Gigg M. A., Lynch V. E., Markvardsen A., Mikkelson D. J., Mikkelson R. L., Miller R., Palmen K., Parker P., Passos G., Perring T. G., Peterson P. F., Ren S., Reuter M. A., Savici A. T., Taylor J. W., Taylor R. J., Tolchenov R., Zhou W., Zikovsky J. (2014). Nucl. Instrum. Methods Phys. Res., Sect. A.

[cit13] Larson A. C., Dreele R. B. V., Los Alamos National Laboratory Report LAUR 86-748, 1994.

[cit14] Finger L. W., Kroeker M., Toby B. H. (2007). J. Appl. Crystallogr..

[cit15] Inoue M., Negishi H. (1986). J. Phys. Chem..

[cit16] Wan C., Wang Y., Wang N., Norimatsu W., Kusunoki M., Koumoto K. (2011). J. Electron. Mater..

[cit17] Kawasaki T., Ohshima K.-I. (2011). J. Phys. Soc. Jpn..

[cit18] Kosuge K., Chemistry of Non-Stoichiometric Compounds, Oxford University Press, Oxford, 1993.

[cit19] Danot M., Brec R. (1975). Acta Crystallogr., Sect. B: Struct. Crystallogr. Cryst. Chem..

[cit20] Danot M., Rouxel J. (1970). C. R. Hebd. Seances Acad. Sci..

[cit21] Danot M., Rouxel J., Gorochov O. (1974). Mater. Res. Bull..

[cit22] Inoue M., Matsumoto M., Negishi H., Sakai H. (1985). J. Magn. Magn. Mater..

[cit23] Neel L., Benoit R. (1953). C. R. Hebd. Seances Acad. Sci..

[cit24] Goodenough J. B., Magnetism and the Chemical Bond, Wiley, New York, 1963.

[cit25] Miyahara S., Teranishi T. (1968). J. Appl. Phys..

[cit26] Reshak A. H., Auluck S. (2003). Phys. Rev. B: Condens. Matter Mater. Phys..

[cit27] Fang C. M., de Groot R. A., Haas C. (1997). Phys. Rev. B: Condens. Matter Mater. Phys..

[cit28] Klipstein P. C., Bagnall A. G., Liang W. Y., Marseglia E. A., Friend R. H. (1981). J. Phys. C: Solid State Phys..

[cit29] Beaumale M., Barbier T., Breard Y., Hebert S., Kinemuchi Y., Guilmeau E. (2014). J. Appl. Phys..

[cit30] Maignan A., Guilmeau E., Gascoin F., Breard Y., Hardy V. (2012). Sci. Technol. Adv. Mater..

[cit31] Ohta M., Obara H., Yamamoto A. (2009). Mater. Trans..

[cit32] Ohta M., Hirai S., Kuzuya T. (2011). J. Electron. Mater..

[cit33] Zhao L.-D., Lo S.-H., He J., Li H., Biswas K., Androulakis J., Wu C.-I., Hogan T. P., Chung D.-Y., Dravid V. P., Kanatzidis M. G. (2015). J. Am. Chem. Soc..

[cit34] Corps J., Vaqueiro P., Aziz A., Grau-Crespo R., Kockelmann W., Jumas J.-C., Powell A. V. (2015). Chem. Mater..

[cit35] Guélou G., Powell A. V., Vaqueiro P. (2015). J. Mater. Chem. C.

