# Supplementary material for: The impact of charge transfer and structural disorder on the thermoelectric properties of cobalt intercalated TiS2
Source: J Mater Chem C Mater. 2016 Feb 2;4(9):1871–80. doi: 10.1039/c5tc04217h (PMC5059785; doi:10.1039/c5tc04217h)
Supplement: Supplementary file 1 [file TC-004-C5TC04217H-s001.pdf]

Electronic Supplementary Material (ESI) for Journal of Materials Chemistry C.
This journal is © The Royal Society of Chemistry 2016

Supplementary Information for:

The Impact of Charge Transfer and Structural Disorder on the Thermoelectric Properties of Cobalt Intercalated TiS_2

Gabin Guélou,[a] Paz Vaqueiro,[a] Jesús Prado-Gonjal,[a] Tristan Barbier,[b] Sylvie Hébert,[b] Emmanuel Guilmeau,[b] Winfried Kockelmann,[c] and Anthony V. Powell*[a]

[a]Department of Chemistry, University of Reading, Whiteknights, RG6 6AH
[b]Laboratoire CRISMAT, UMR6508 CNRS ENSICAEN, 6 bd Marechal Juin, 14050 Caen Cedex 4, France
[c]ISIS Facility, Rutherford Appleton Laboratory, Chilton, Oxfordshire OX11 0QX

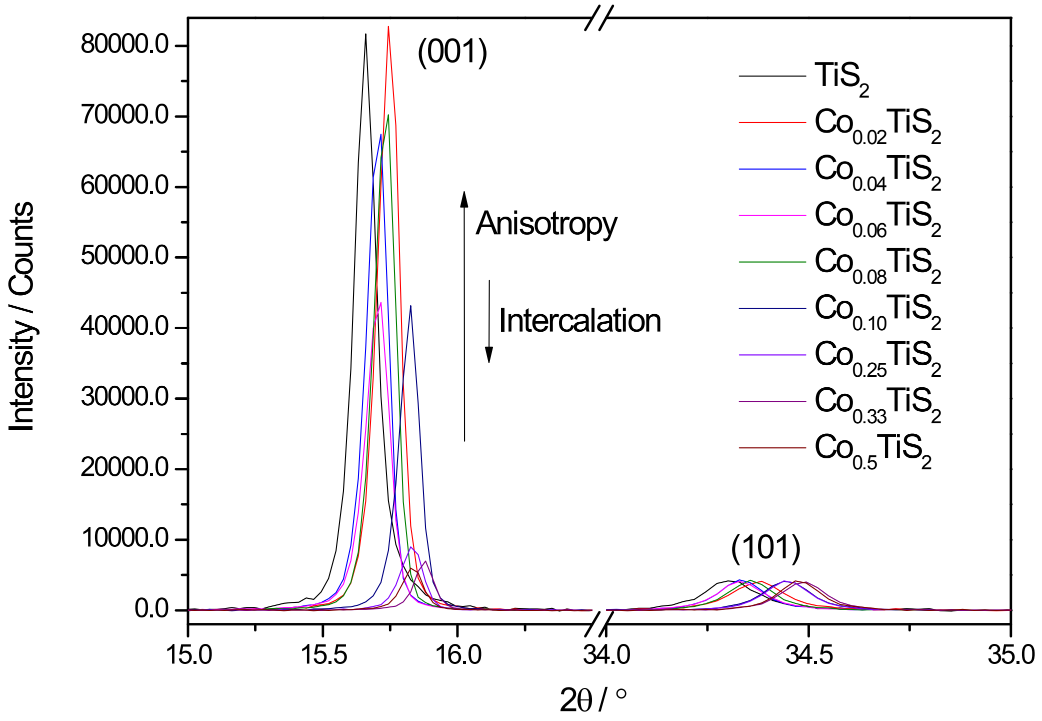

Figure S1 Superimposed XRD plots taken from consolidated Co_xTiS_2 pellets highlighting the change in the ratio of the (001) and (101) reflections due to variations in the degree of preferred orientation.

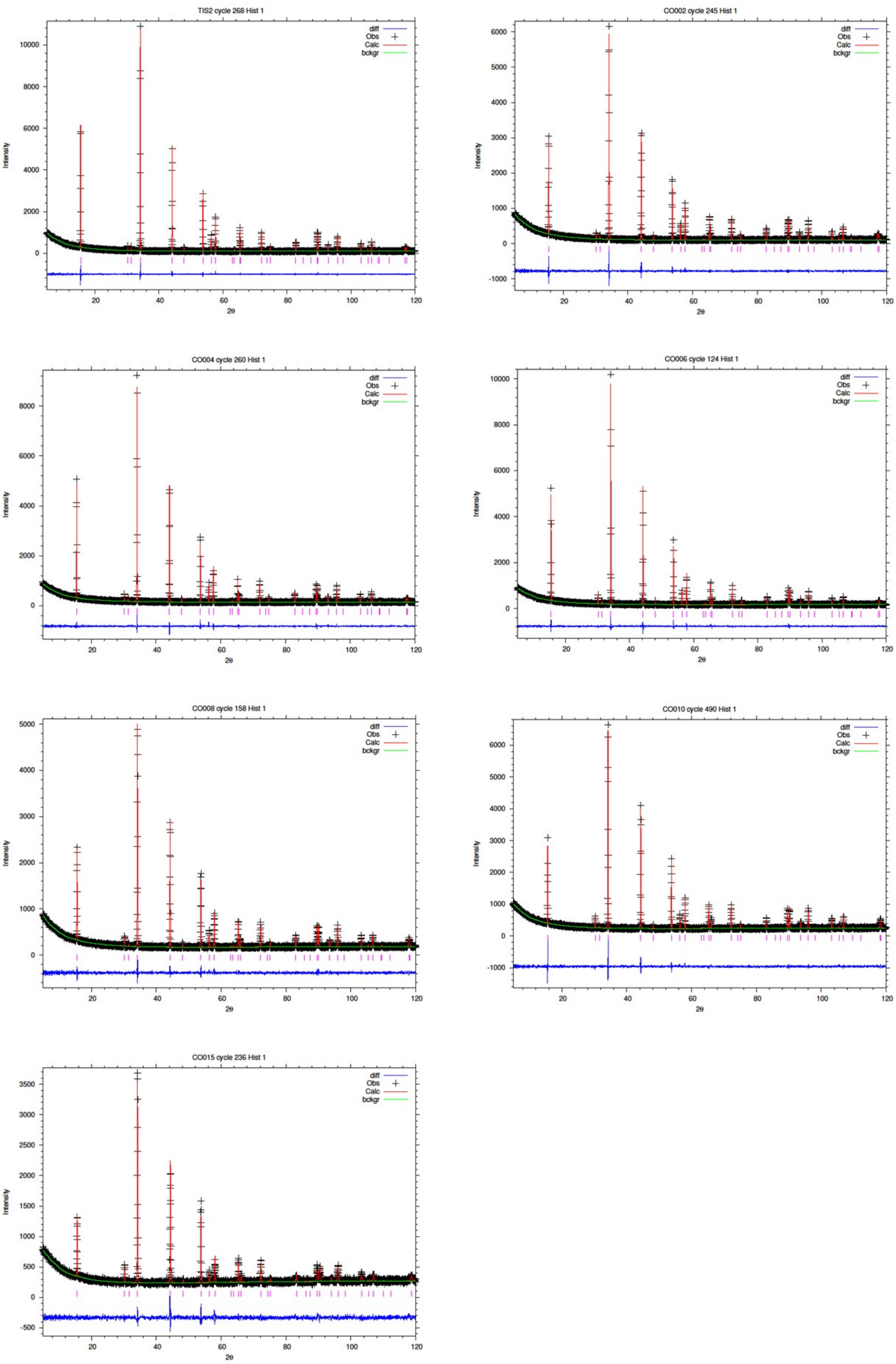

Figure S2 Observed (crosses), calculated (red line), calculated background (green line) and difference profiles (bottom blue line) for Co_xTiS_2 from Rietveld refinement using powder X-ray diffraction data (reflection positions are marked). From left to right and top to bottom: x = 0, 0.02, 0.04, 0.06, 0.08, 0.10 and 0.15.

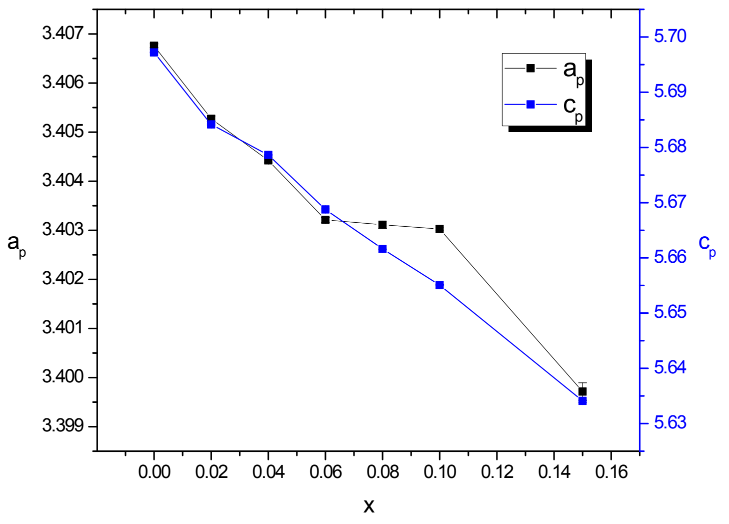

Figure S3 Lattice parameters of Co_xTiS_2 ($0 \leq x \leq 0.15$) as a function of x, determined from powder X-ray diffraction data.

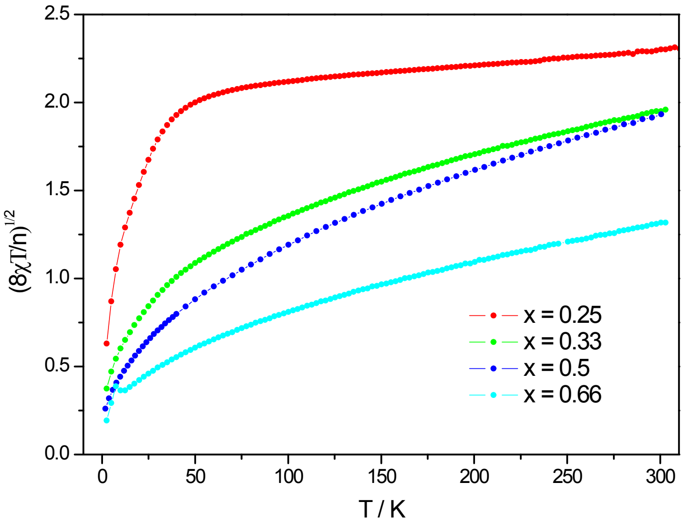

Figure S4 Temperature dependence of the effective magnetic moment per cation of Co_xTiS_2, as measured by the quantity $(8\chi T/n)^{1/2}$.

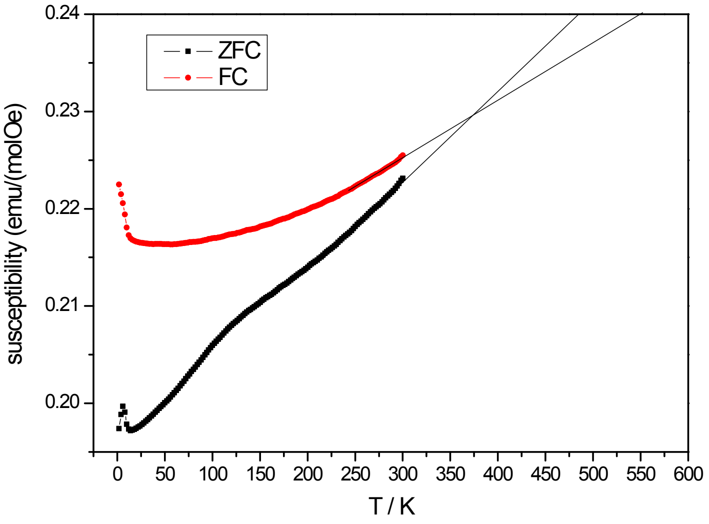

Figure S5 Zero-field cooled (black squares) and Field-cooled (red circles) molar magnetic susceptibility of $Co_{0.75}TiS_2$

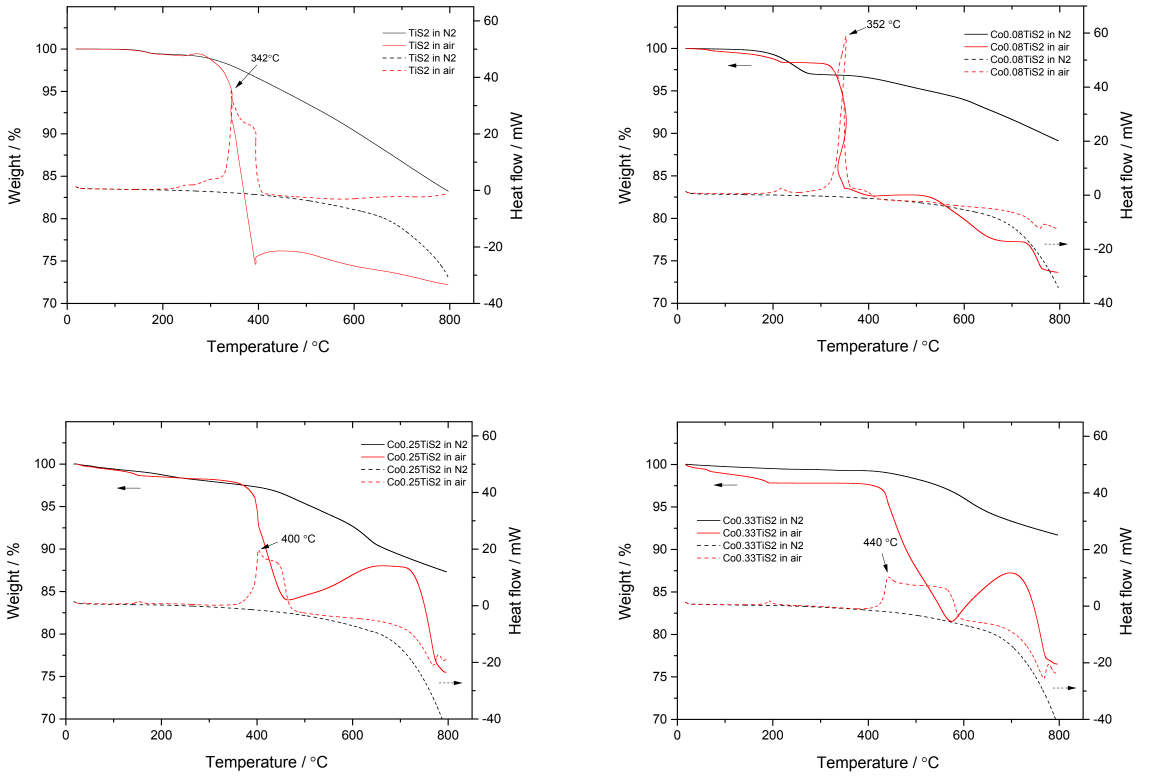

Figure S6 TGA/DSC data for Co_xTiS_2 ($0 \leq x \leq 0.33$). Full lines correspond to weight loss and dashed lines to heat flow.

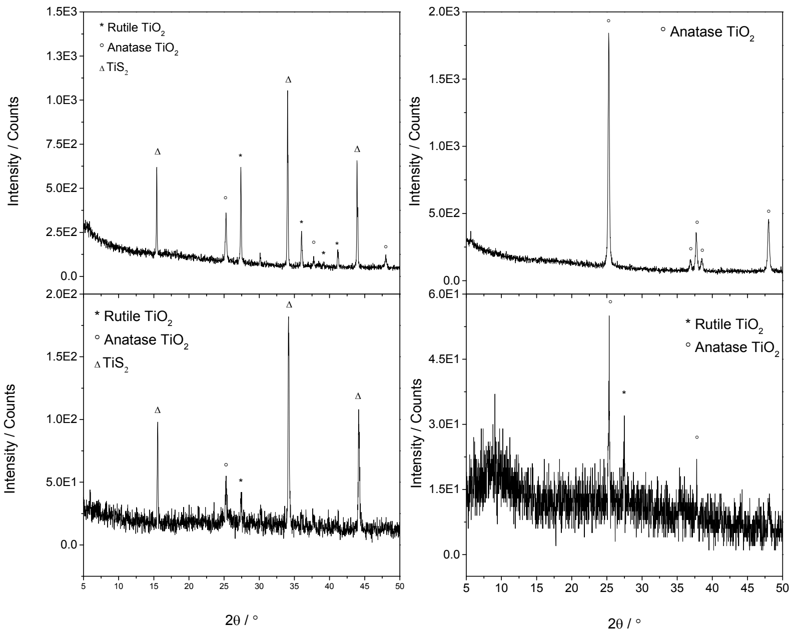

Figure S7 Powder X-ray diffraction data for the products of thermogravimetric analysis of Co_xTiS_2 (top: x = 0; bottom: x = 0.08) up to 800°C in N_2 atmosphere (left) and in air (right).

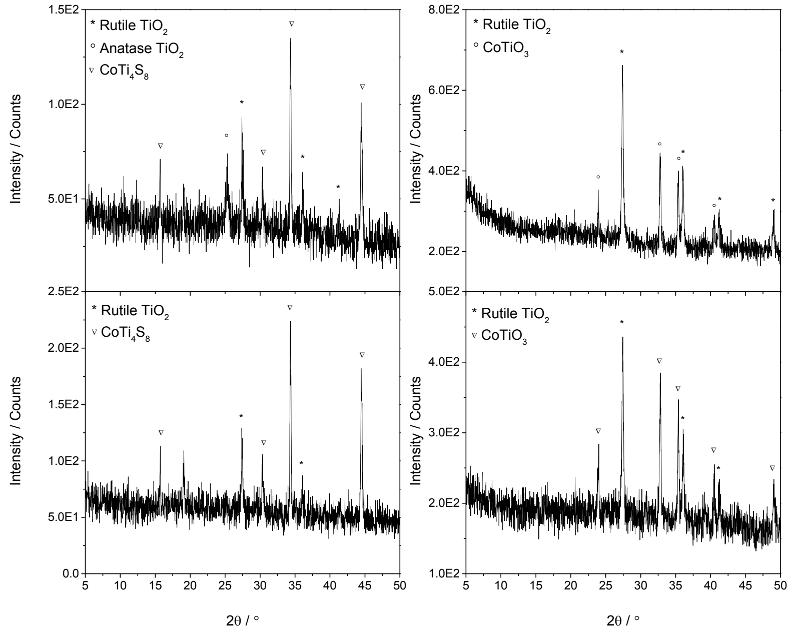

Figure S8 Powder X-ray diffraction data for the products of thermogravimetric analysis of Co_xTiS_2 (top: x = 0.25; bottom: x = 0.33) up to 800°C in N_2 atmosphere (left) and in air (right).

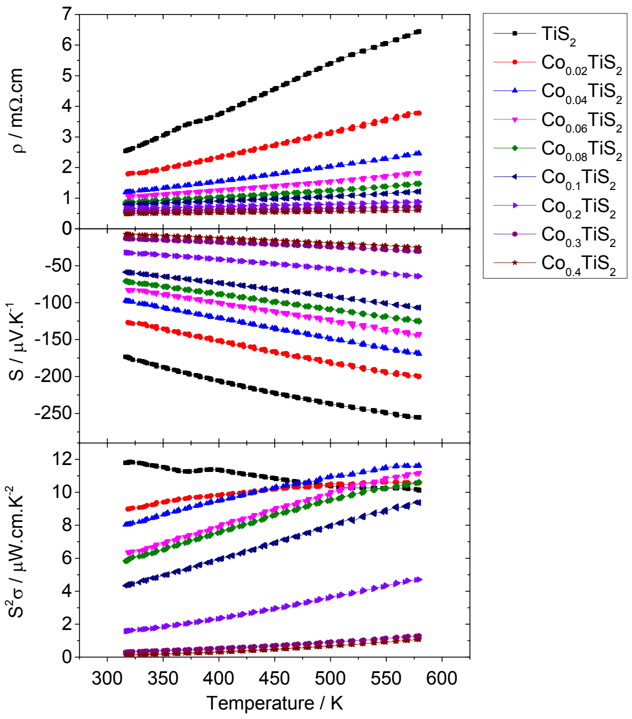

Figure S9 Measurements of the temperature dependence of the electrical resistivity, Seebeck coefficient and power factor of along the in-plane direction for non-ball milled samples of Co_xTiS_2 ($0 \leq x \leq 0.4$).

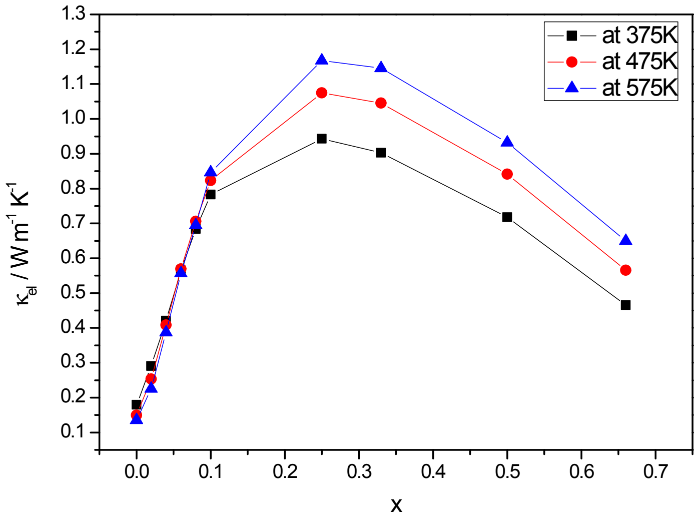

Figure S10 Electronic contribution to the in-plane thermal conductivity in Co_xTiS_2 ($0 \leq x \leq 0.66$) as a function of cobalt intercalation, x, at 3 selected temperatures determined using the Wiedemann-Franz law

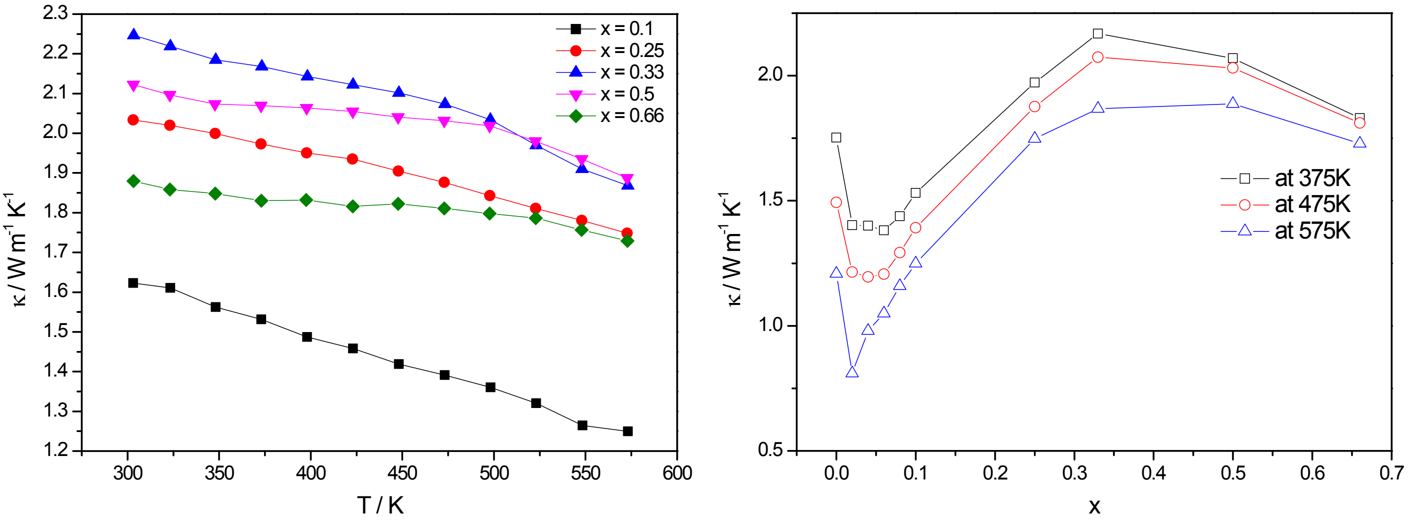

Figure S11 Temperature dependence of the cross-plane thermal conductivity of Co_xTiS_2 ($0 \leq x \leq 0.66$) (left) and its evolution with composition at three selected temperatures (right).
